# Investigation of Mechanical Properties and Microstructural Characteristics of Earth-Based Pavements Stabilised with Various Bio-Based Binders

**DOI:** 10.3390/polym17070864

**Published:** 2025-03-24

**Authors:** Nuriye Kabakuş, Yeşim Tarhan

**Affiliations:** 1 Department of Emergency and Disaster Management, Faculty of Applied Sciences, Atatürk University, Erzurum 25240, Türkiye; 2 Technical Sciences Vocational School, Ardahan University, Ardahan 75000, Türkiye; yesimtarhan@ardahan.edu.tr

**Keywords:** earth roads, biobased materials, xanthan gum, new-generation pavement

## Abstract

For centuries, earthen materials have regained popularity because of the high carbon emissions caused by the construction sector. Although earth-based materials possess superior properties, such as recyclability, easy accessibility, affordability, and high thermal conductivity, they are not without drawbacks. They are, for instance, relatively weak and sensitive to water, and their physical and chemical properties can vary considerably depending on the source from which they are obtained. Stabilisation is often used to overcome these drawbacks. In this study, natural earth-based materials were stabilised with biopolymers of organic origin, such as alginate, Arabic gum, xanthan gum, and locust bean gum, to preserve their natural properties. To produce the samples, the earth material used in the road sub-base layer was mixed with kaolin clay and silica sand, and the mixtures were prepared by substituting biopolymer materials with clay at a ratio of 0.1%. After determining the fresh unit volume weights, spreading diameters (flow table test), penetration depths (fall cone test), and air content of the mixtures, the flexural and compressive strengths of the cured specimens were measured. In addition, scanning electron microscopy (SEM) and X-ray diffraction (XRD) analyses were performed to determine the microstructural characteristics. According to the 28-day compressive strength results, the mix with xanthan gum was found to be almost twice as strong as the other mixes. It has been concluded that biopolymer-stabilised earth mixtures can be used as a fill material in buildings where high strength is not required, or as a paving material on low-traffic roads.

## 1. Introduction

Earth-based natural materials have been used widely in the construction industry since ancient times [1]. The use of energy-intensive materials, such as cement and bitumen, in this sector has increased environmental problems. With growing interest in sustainable building materials, earthen mixtures have once again come to the fore [2]. Currently, scientists are exploring various methods to enhance the strength and durability of earth-based mixtures, including stabilization techniques [3,4,5] and fiber reinforcement [6]. Additionally, advancements in construction technologies, such as 3D printing, are gaining traction due to their potential to optimise material usage, reduce waste, and incorporate sustainable materials like recycled plastics and bio-based composites, thereby contributing to circular economy principles and sustainable building practices [7,8,9,10]. The increasing demand for sustainable building materials has accelerated efforts to standardise earth-based mixtures’ design, application, and testing. Earth-based materials are often used in the construction of unpaved roads [11,12,13], road sub-base layers [14], cycle paths, pedestrian roads [12], berms, and some non-high-strength structures [15].

As demand increases, studies and strategies are being developed to replace depleted petroleum resources with renewable resources that offer environmental and economic benefits. In this context, the new goal in pavement engineering is to reduce the use of bituminous materials and emphasise the use of renewable resources, such as biopolymers. Biopolymers are preferred binders for earth-based materials because they are natural materials derived from plants, animals, and microorganisms, have a low environmental impact, and contribute to strength. These materials have been successfully used in the construction sector to stabilise various building and road materials.

The stabilisation of earth-based materials with biopolymers is an emerging field, driven by the need for sustainable construction practices and the reduction in environmental impacts associated with traditional stabilisers, such as cement and lime [16,17]. Biopolymers, which are environmentally friendly and derived from natural sources, offer a promising alternative with the potential to improve the mechanical properties of earth structures while reducing carbon emissions [18]. However, the use of biopolymers is challenging and still needs to be improved. Poor water resistance and susceptibility to biodegradation can limit their effectiveness and durability [19]. Research is ongoing to improve these properties through novel treatments, such as acetylation and the addition of inorganic minerals. Studies have also shown that biopolymers, such as casein and sodium caseinate, can significantly improve the compressive strength of treated sand, with factors, such as curing time and temperature, playing a role in the stabilisation process [16,20].

The stabilisation of the earth is one of the primary methods for correcting the expanding earth in response to moisture changes and improving its engineering quality [20]. Although there are many studies on soil stabilisation materials and techniques in the literature, there are only a limited number of studies on stabilisers (especially biopolymers) and techniques recommended for earth-based pavements [21,22].

This study developed an earth-based unpaved road mix using biopolymers as binders as an alternative to flexible and rigid pavements that use bitumen and cement as binders for low-volume road pavements. The engineering properties of mixtures obtained by adding biopolymers, which are also natural binders, to natural and locally available earth and clay mixtures were investigated. The biopolymers used in this study, which investigated the most effective type of biopolymer that could increase the strength and durability of this earth-based mix, were selected from water-soluble gums [23]. Arabic gum (AG) from the resin group, alginate (A) from seaweed extracts, locust bean gum (LBG) from seed extracts, and xanthan gum (KG) from microbial gums were used. In this way, unlike studies in literature, the effect of different categories of gums in earth-based mixtures was investigated.

The water solubility of biopolymers is a key factor influencing their performance as earth-stabilising agents. When dissolved in water, these biopolymers form hydrogels or viscous solutions that enhance soil cohesion, modify rheological properties, and improve mechanical stability [24]. Their capacity to interact with soil particles and pore water enables them to function as binding agents, thereby reducing erosion, regulating moisture fluctuations, and increasing soil strength [25]. Among water-soluble biopolymers, xanthan gum, guar gum, alginate, and Arabic gum have demonstrated significant potential for soil stabilisation due to their capacity to form cross-linked polymer networks. In particular, xanthan gum and guar gum create three-dimensional hydrogel structures that improve the mechanical properties and durability of soil, making them highly suitable for road stabilisation [26]. Xanthan gum, in particular, has been shown to enhance water retention, reduce evaporation and percolation, and mitigate soil erosion—critical factors in maintaining road stability [23].

This study investigates the potential of biopolymer stabilisation in soil mixes by incorporating four types of biopolymers—alginate, gum arabic, xanthan gum, and locust bean gum—into a road sub-base soil mix with sand and kaolin clay. The primary objective is to evaluate the feasibility of these stabilised mixtures as paving materials for low-traffic roads. Fresh state properties were assessed and hardened specimens were tested for mechanical strength and durability. In addition, the microstructural analysis provided insight into the stabilisation mechanisms. The results contribute to a broader understanding of biopolymer-stabilised soil materials and their role in sustainable road construction.

## 2. Materials and Methods

### 2.1. Materials and Specimen Preparation

**Figure 1:**
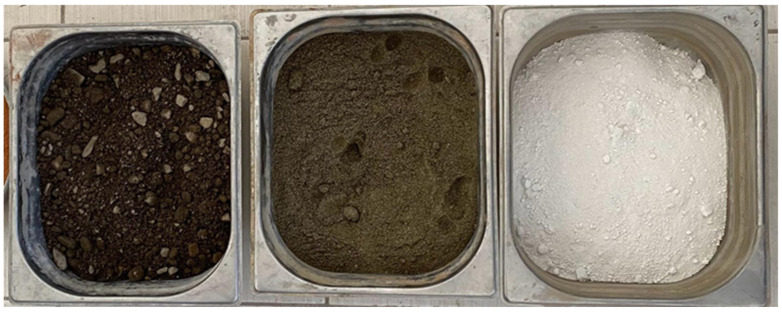
Road subgrade earth, sand, and kaolin clay, from left to right.

In this study, a mixture of soil, sand, and clay stabilised with four types of biopolymers to determine the most effective biopolymer for enhancing the mechanical performance of an earth-based mixture. The primary soil used in the mixture was classified as silty sand (SM) according to the Unified Soil Classification System (USCS), with a unit volume weight of 2.35 g/cm^3^. This material, sourced from a road sub-base layer, was crushed and sieved to a 0–2 mm particle size to ensure uniformity. The silica sand component, with a bulk density of 2.47 g/cm^3^, was included in the 0–1 mm size range to enhance gradation. Kaolin clay, supplied by ATA KİMYA (the company in Ankara, Türkiye) commercial limited company as a dry, ground material packaged in 20 kg bags, was incorporated as binder. Figure 1 presents images of all raw materials used in this study.

The grain diameter distribution and chemical composition of kaolin clay were provided by ATA KİMYA (the company in Ankara, Türkiye), while the geotechnical properties were determined through experimental tests conducted at the Soil Mechanics Laboratory of Atatürk University’s Faculty of Engineering. Hydrometer analysis confirmed that 100% of kaolin particles passed through the 0.075 mm sieve. The chemical composition, analysed via X-ray fluorescence spectrometry (XRF), and the geotechnical properties, obtained from both laboratory experiments and the manufacturer’s published data, are presented in Table 1.

Alginate (A), Arabic gum (AG), xanthan gum (KG), and locust bean gum (LBG) were added to the control mixture (C) to stabilise the earth-based mixture. The properties of the biopolymers are listed in Table 2.

Dry mixes were prepared using 40% by weight road sub-base, 30% sand, and 30% clay. Tap water was added to 20% of the total mix to ensure a suitable consistency for workability in the control mix. To stabilise the mix, biopolymers were incorporated at 0.1% by weight of clay, proportioned to the total clay content. The biopolymers were first dissolved in water to form a slightly gel-like consistency before being incorporated into the dry mix.

In preliminary trials, the biopolymer ratio was initially set at 1% of the clay content, but this resulted in excessive gelation, making the mixture too dense and highly viscous, preventing proper mixing with the dry components. A subsequent 0.5% substitution was tested, but the mix still had a solidified consistency that prevented proper moulding. Based on these observations, a 0.1% biopolymer level was selected as it provided a workable consistency that allowed for proper mixing and moulding. The final compositions of the mixes used in this study are shown in Table 3.

A 5-litre laboratory mortar mixer was used to prepare the mixtures. First, the dry components were mixed for 1 min, then the biopolymer solution—prepared by dissolving the biopolymer in water—was added. The mixture was then mixed at low speed for 1 min, followed by a 1 min rest period, and finally mixed at high speed for 2 min. The biopolymers were first dissolved in water for 2 min using a magnetic mixer before being incorporated into the soil mixture.

For mechanical testing, 4 × 4 × 16 cm^3^ beam specimens were prepared for flexural strength evaluation, while 5 × 5 × 5 cm^3^ cube specimens were cast for compressive strength testing. Due to the gel-forming nature of biopolymers, specimens were manually placed in moulds rather than compacted, as immediate compaction could result in uneven density distribution and affect mechanical performance. The samples were left undisturbed for 1 day at 21 ± 2 °C and 50% relative humidity under laboratory conditions. After demoulding, specimens were oven-dried at 60 °C for 1 day to ensure controlled moisture removal and to maintain consistency across all specimens. This temperature was chosen to prevent excessive shrinkage while promoting uniform drying. Whilst thermal drying can increase the compressive strength of clay containing materials, the effect was consistent across all mixtures tested, ensuring reliable comparisons. The samples were then cured for 28 days at 23 ± 2 °C and 65% relative humidity to allow further strength development under controlled conditions.

### 2.2. Experimental Program

The unit volume weights, air content [29], spreading diameter (measured using a flow table test to evaluate the viscosity and workability of the mixtures) [30], and penetration depth (measured using a fall cone test to determine the thixotropic behaviour of the mixtures) [31] were evaluated. The flow table test, typically used for cementitious materials, was used in this study to evaluate the workability of biopolymer-stabilised mixtures. Due to the gel-forming nature of biopolymers, conventional soil workability tests do not adequately capture the changes in mix consistency. The flow table test provided a controlled assessment of the flowability and deformation characteristics of the mixes, ensuring a standardised comparison between different formulations.

The cured specimens were subjected to three-point flexure at 28 days and uniaxial compression tests at 28 and 56 days to evaluate their mechanical performance over time. The loading rate was set at 0.004 MPa/s for the flexure test and 0.04 MPa/s for the compression test.

**Figure 2:**
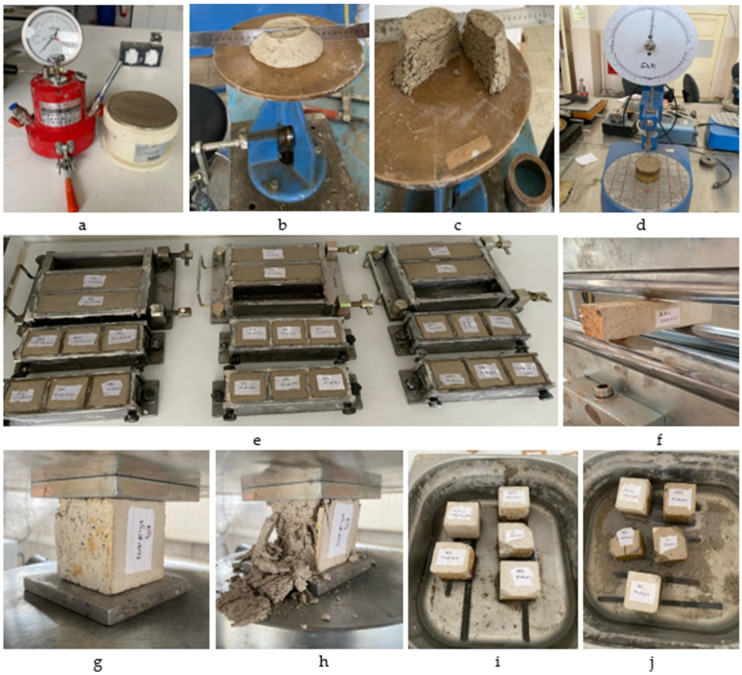
Tests applied: (**a**) Air content determination; (**b**,**c**) Flow table; (**d**) Fall cone; (**e**) Sample preparation; (**f**) Flexural strength; (**g**,**h**) Compressive strength; and (**i**,**j**) Capillary water absorption.

Capillary water absorption tests were performed to assess the durability of the samples according to the methodology outlined in [32]. This test evaluates the water absorption capacity of the material over time, expressed as a percentage increase in mass relative to the initial dry weight. To ensure consistency, the samples were dried in an oven at 40 ± 2 °C for seven days until a stable mass was reached, maintaining a uniform moisture content prior to testing. After drying, the sides of the samples were sealed with tape to prevent lateral water absorption. The capillary water absorption test was carried out by placing the samples on steel rods in a tray, ensuring that only the lower surface (up to a height of 5 mm) was in contact with tap water. Water uptake was recorded as a percentage increase in mass relative to the initial dry weight to ensure consistency with standardised reporting methods. Results are presented as percentage mass gain (%) to allow comparability with similar studies. Pictures of the test set-up are shown in Figure 2.

After 28 days of curing, all mechanical tests, including evaluation of compressive and flexural strength, were performed. However, SEM and XRD analyses were carried out on specimens taken from the specimens after 56 days. These analyses were carried out at Atatürk University DAYTAM to investigate the microstructural properties. SEM images were obtained using a Zeiss Sigma 300 scanning electron microscope. XRD analyses were performed using a Malvern PANalytical EMPYREAN X-ray diffractometer (manufactured in Almelo, The Netherlands) with Cu-α radiation (λ = 1.54 Å) on an X-ray diffractometer. The scan range (2θ) was 10–90° with a scan speed of 4°/min, an operating voltage of 5 kV, and an operating current of 40 mA.

## 3. Results and Discussion

### 3.1. Fresh State Results

**Figure 3:**
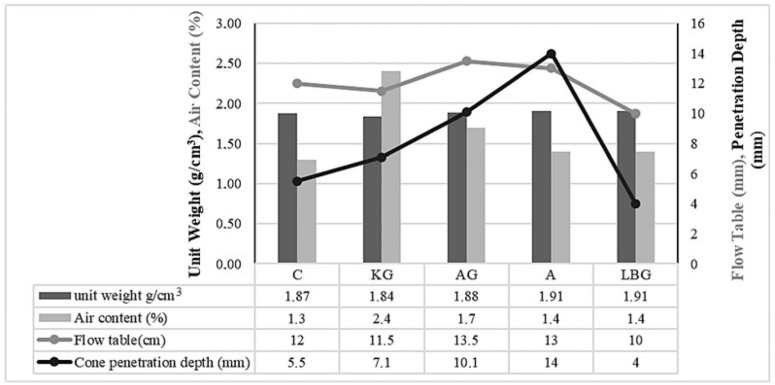
Fresh-state test results.

This study investigated the potential of stabilising earth-based construction materials with biopolymer materials to improve their mechanical properties. The results of the fresh unit volume weight, air content, spreading diameter, and penetration depth tests of the mixtures are plotted in Figure 3. In the graph in Figure 3, all fresh-state test results are plotted together for comparison.

Figure 3 shows that the unit volume weights of all mixes are below 2 g/cm^3^, and the values show a very close distribution. In this study, the mix with the lowest unit volume weight and the highest air content was observed in the xanthan gum-stabilised group. In terms of penetration values, the highest penetration depth was obtained in the group with alginate, while the lowest value was recorded in the group with locust bean gum. Based on the flow table results, the lowest flow value was recorded in the group in which locust bean gum was added, indicating that the mixture to which locust bean gum was added had a more solid-like consistency and, therefore, a higher unit volume weight compared to the other mixtures. Thus, the falling cone and flow table experiments supported each other. As can be seen from the graph, the results from the flow table and the penetration depth are generally in agreement, indicating that these two tests are effective tools for determining the rheological properties of the mixtures, that is, their consistency. Mixing biopolymers with water resulted in a gel-like consistency, increasing the viscosity of the mixtures, and consequently resulting in a more solid structure.

### 3.2. Hardened State Results

**Figure 4:**
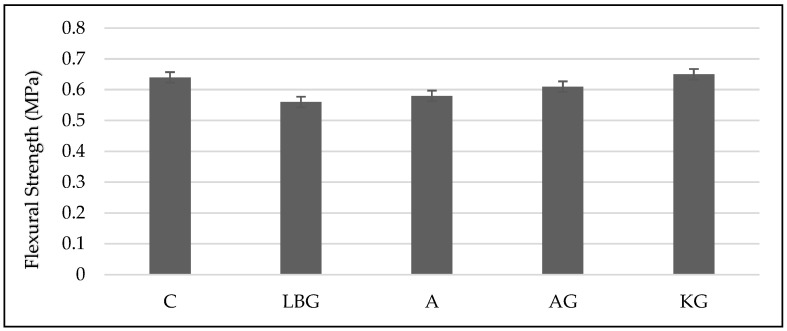
Flexural strength test results.

The 28-day flexural strengths of the control group and all the groups to which the biopolymer stabiliser was added are shown in Figure 4.

Analysis of the flexural strength results (Figure 4) shows that the highest flexural strength was achieved in the KG group, reaching 0.65 MPa. The stabilisation process with KG increased the flexural strength of the earth-based specimens by approximately 2%. Although this increase may seem small, even small improvements are significant given the inherently brittle nature of earth-based materials.

In contrast, biopolymers other than KG had a negative effect on flexural strength. Among the formulations tested, the KG group exhibited the highest consistency, as evidenced by its low unit volume weight, high air content, and cohesive behaviour. As can be seen in Figure 2c, the KG mix exhibited superior consistency compared to the other formulations, despite its lower unit volume weight. This suggests that KG improves the bonding between the soil materials, contributing to higher flexural strength.

The flexural strength test results support this observation, showing that the KG group had a greater flexural strength capacity than the other groups. This finding reinforces the conclusion that KG acts as an effective binder, improving both cohesion, and mechanical performance.

**Figure 5:**
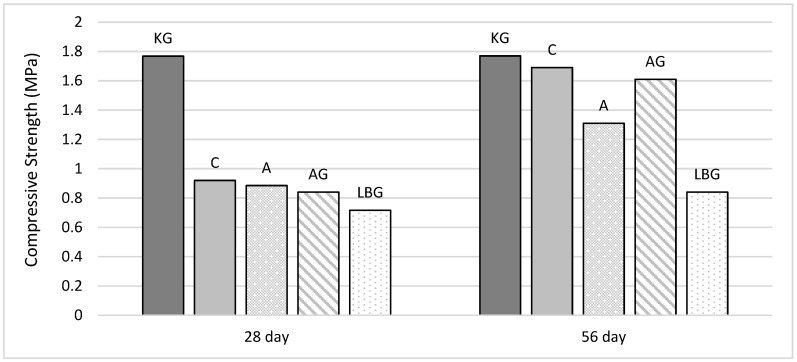
Compressive strength test results for 28 and 56 days.

The 28 and 56-day compressive strength values of the earth-based specimens are shown in Figure 5. In addition, the increasing trends representing the relationship between the specimens in the figure are shown separately for the 28-day and 56-day compressive strength results.

According to the results presented in Figure 5, the compressive strength at 56 days increased across nearly all groups compared to 28 days, indicating that the soil-based specimens continued to gain strength over time. This increase was particularly pronounced in the control group without biopolymers, which exhibited an 84% strength increase compared to 28 days. The increase in strength is likely due to densification, im-proved interparticle bonding, and void reductions over time, which have been identified in soil mechanics studies as key contributors to the strength development of soil-based materials [33,34,35,36]. Studies show that biopolymers, such as chitosan, xanthan gum, and guar gum, improve interparticle bonding and significantly increase soil strength [37,38]. For example, the chitosan biopolymer increased the compressive strength of sand by up to 320 kPa and improved the cohesion by 34.2 kPa [38], while the combination of xanthan gum and guar gum resulted in a greater shear strength than individual treatments [39].

The KG group exhibited the highest compressive strength at both 28 and 56 days, with almost identical values at both time points. This suggests that xanthan gum promoted early strength development by improving cohesion, water retention, and interparticle bonding through its hydrogel-forming ability. Unlike hydraulic binders, such as cement, xanthan gum does not undergo hydration reactions but instead forms a three-dimensional polymeric network that fills soil pores, improves load distribution, and reduces voids, leading to improved mechanical performance. Its hydrogel formation plays a critical role in soil stabilisation by cementing soil particles together, significantly improving inter-particle cohesion [40,41]. Studies have shown that even a 1% xanthan content can increase cohesion by 3.8 to 14 times compared to untreated soil [42]. In addition, its water-retaining properties help to retain moisture in the soil pores, further contributing to its stabilising effect [43].

In contrast, the LBG group exhibited the lowest compressive strength, even lower than the control group, indicating that LBG was ineffective in improving the strength of earth-based mixtures. Cheng and Geng [44] compared the effectiveness of different biopolymers in improving the unconfined compressive strength (UCS) of clay and found that sodium alginate provided the highest UCS improvement, while LBG was not specifically identified as an effective stabiliser.

The AG (Arabic gum) group showed a gradual increase in strength, reaching values close to those of the control group at 28 days and approximately 90% of its maximum strength at 56 days. However, as its compressive strength remained lower than that of the control mix, it cannot be classified as a strength-enhancing binder. While Brzyski [45] reported that Arabic gum at concentrations of 3% and 5% significantly increased the flexural strength (by ~300%) and compressive strength (by 25% and 60% respectively) in lime–metakaolin pastes, the results of this study suggest that the effectiveness of AG as a binder is limited for the soil-based mixtures tested. Similarly, the A group (alginate) showed a 30% increase in strength at 56 days compared to 28 days, indicating that while it improved in strength over time, its performance was less effective than that of the AG group.

The earth-based samples were subjected to capillary water absorption tests for durability testing. The results of the capillary water absorption tests are presented in Table 4.

During the capillary water absorption test, the KG and LBG samples continued to gain weight due to water absorption after immersion, while the C, A, and AG samples began to dissolve and lose weight within minutes.

For the KG and LBG samples, capillary water reached the top surface within 30 min, and both remained intact during this period. However, after 30 min, they began to disperse, and the test was terminated. Despite the negative effect on mechanical properties, locust bean gum (LBG) exhibited temporary resistance to water penetration before eventually disintegrating. The poorest water resistance was observed in the AG group, where samples disintegrated and lost material immediately upon immersion.

KG improved both mechanical properties and durability, improving also resistance to water absorption.

### 3.3. Micro Structural Analysis Results

**Figure 6:**
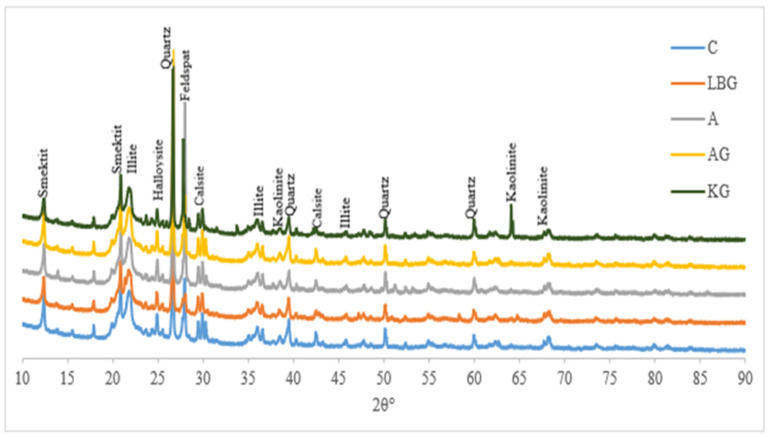
XRD graphs of earth-based mixtures stabilised with biopolymers.

The graphs obtained from the XRD analysis of the samples taken after the 56-day compressive strength test are shown in Figure 6.

The XRD analysis of the mixtures (Figure 6) revealed the presence of ceramic-based minerals, including quartz, kaolinite, feldspar, smectite, and illite, with no significant differences observed between the control and biopolymer-modified samples. The absence of biopolymer-related peaks in the XRD spectra is consistent with the findings in the literature, which highlight that XRD primarily detects crystalline structures and has a limited sensitivity to non-crystalline or amorphous organic compounds [46]. Research has demonstrated that biopolymers introduced at low substitution rates (≤1%) frequently fall below the detection threshold of XRD due to their weak diffraction signals and amorphous nature [47,48].

Furthermore, the presence of biopolymers may be diminished by high-energy irradiation, thereby further reducing their detectability in XRD analysis [37]. Despite their absence in XRD spectra, biopolymers have been documented to improve soil cohesion and mechanical performance through physical interactions, such as hydrogel formation, interparticle bonding, and moisture retention, rather than through crystalline phase alterations [39,40,49].

These enhancements occur without compromising the crystalline structure of soil minerals. Instead, biopolymers form fibrous and reticulated networks that fill voids, bond soil particles, and enhance interparticle cohesion, leading to increased strength, water stability, and erosion resistance [40].

**Figure 7:**
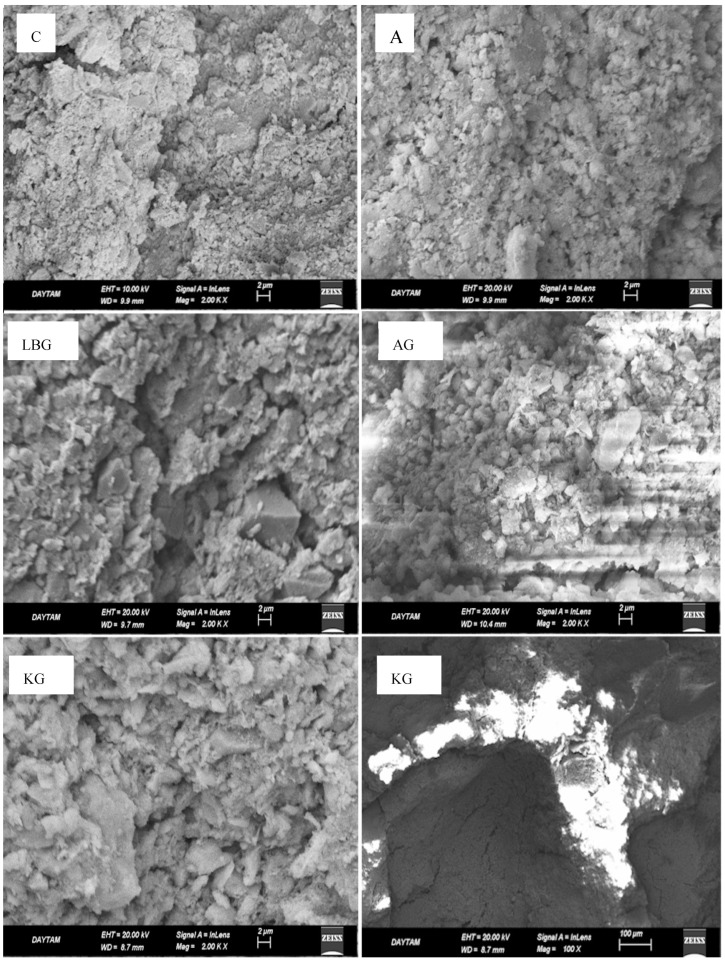
SEM images of the mixtures.

The SEM images used to visualise the microvoids and bond structures formed in the control, LBG, A, AG, and KG group samples are shown in Figure 7.

The SEM analysis (Figure 7) confirmed the presence of clay minerals in all mixtures. However, due to the low substitution rate (0.1%) of biopolymers, no pronounced biopolymer bonds with earth-based materials were observed. This finding supports the XRD and compressive strength results, suggesting that the biopolymers affected soil properties through physical rather than chemical interactions.

The LBG and KG groups exhibited a more crystalline mineral structure, while other groups exhibited a more reticulated morphology. A closer examination of the KG sample at 100× magnification (lower right-hand corner of Figure 7) revealed large capillaries and macrovoids, a feature observed in all groups. The high percentage of voids was identified as a key factor contributing to the poor mechanical and durability performance of the earth-based mixes.

## 4. Conclusions

This study investigated the potential for improving the mechanical properties of earth-based building materials by stabilising them with four types of biopolymer materials (xanthan gum, Arabic gum, alginate and locust bean gum).

The addition of the biopolymer reduced the unit volume of the mixture. The mixtures stabilised with xanthan gum reached a more solid consistency despite having a low unit volume weight and high air content. When biopolymers were mixed with water, they changed to gel consistency and increased the viscosity of the mixtures.

The incorporation of biopolymers improved both flexural and compressive strength, demonstrating their potential as binders in soil-based materials. Among the formulations tested, xanthan gum exhibited the highest strength values at both 28 and 56 days and showed the greatest resistance to capillary water absorption.

Microstructural analyses (XRD and SEM) confirmed the presence of crystalline minerals but did not detect biopolymer components, probably due to their non-crystalline nature and low substitution rate (0.1%). While the SEM analysis revealed differences in bonding structures and the presence of macrovoids, further investigation is needed to better understand biopolymer–soil interactions at the molecular level.

Therefore, Fourier Transform Infrared (FTIR) spectroscopy is recommended as it can identify functional groups in biopolymers and detect potential chemical interactions between biopolymer molecules and soil particles that are not captured by SEM or XRD analyses.

In conclusion, this study showed that the mechanical and durability properties of earth-based building materials can be improved when stabilised with biopolymers. Of the biopolymer types investigated in this study, xanthan gum improved the properties of the earth-based mix more than the other biopolymers. Biopolymers can be effective components in the development of sustainable building materials and can potentially reduce environmental impact and improve material performance. Based on the results of this study investigating the effective biopolymer type, it is recommended that future research should investigate the optimum biopolymer substitution ratio and the results of advanced day strengths (90, 180, and 360).

While biopolymer-based stabilisation is an emerging and promising approach to sustainable construction, a universally accepted standard for evaluating its performance has yet to be established. However, this study provides a quantitative assessment of mechanical and durability properties, consistent with previous research on biopolymer-stabilised soils. The results show that xanthan gum significantly improves cohesion, flexural strength, and water resistance, highlighting its potential for soil-based stabilisation. Future research should prioritise the development of standardised test methods to enable wider adoption and comparative analyses within the construction industry.

## Figures and Tables

**Table 1:** Chemical and geotechnical properties of kaolin clay [27,28].

Chemical Properties	Value (%)	Geotechnical Properties	Value (%)
SiO_2_	69.10	Grain size < 0.002 mm, %	28
Al_2_O_3_	15.20	Grain size < 0.075 mm, %	100
Fe_2_O_3_	0.20	Specific gravity	2.63
CaO	0.10	Liquid limit, %	49
SO_3_	4.29	Plastic limit, %	26
Na_2_O	0.03	Plasticity index, %	23
K_2_O	11.07	Optimum moisture content, %	25
CR_2_O_3_	0.01	Maximum dry unit weight, kN/m^3^	13.2
		Unconfined compressive strength, kPa	307

**Table 2:** Properties of biopolymers used.

Product Properties	LBG	A	AG	KG
CAS number	9000-40-2	14984-39-5	9000-01-5	11138-66-2
Appearance/ texture/smell	Pale white, no smell	White, powder, no smell	White, powder, slightly smelly	White, solid, no smell
pH	5.4–7	6.0–8.0	4.1–4.8	6–8
Density	1.2 ± 0.1 g/cm^3^	1.6 g/cm^3^	1.15 g/cm^3^	1.5 g/cm^3^
Molecular weight	226.66 g/mol	216.121 g/mol	180.41 g/mol	1016.8 g/mol
Molecular formula	C_10_H_11_CIN_202_	C_6_H_7_O_6_Na	C_12_H_36_	(C_35_H_49_O_29_)_n_
E-code	E410	E401	E414	E415

**Table 3:** Earth-based mix designs stabilised with biopolymers.

Sample Code	Material Amounts (%)							
	Earth	Sand	Clay	Water	A	AG	KG	LBG
C	40	30	30	20	-	-	-	-
A	40	30	29.9	20	0.1	-	-	-
AG	40	30	29.9	20	-	0.1	-	-
KG	40	30	29.9	20	-	-	0.1	-
LBG	40	30	29.9	20	-	-	-	0.1

**Table 4:** Capillary water absorption test results.

Sample	Beginning	2 min Later Weight (g)	6 min Later Weight (g)	30 min. Later Weight (g)	Mass Change (%)
C	204.19	206.63	206.39	195.39	(−) 4.31
A	188.11	188.76	190.43	170.61	(−) 9.3
LBG	190.62	203.04	206.06	219.12 **	(+) 14.95
AG	198.48	185.44	178.36	*	(−) 10.14
KG	195.60	201.98	203.79	215.33	(+) 10.09

* Disintegrated before leaving water. ** Water level reached top of sample.

## Data Availability

All data supporting the reported results of this study are included within the article.
